# Respuesta con enfoque de derechos de la niñez frente a la pandemia por COVID-19 en Chile, Colombia y Perú

**DOI:** 10.26633/RPSP.2021.151

**Published:** 2021-12-22

**Authors:** Fernando González, María Camila Pinzón-Segura, Bertha Luz Pineda-Restrepo, María del Carmen Calle-Dávila, Estefanía Siles Valenzuela, Natalia Herrera-Olano, María Lucía Mesa Rubio

**Affiliations:** 1 Oficial de Salud UNICEF Chile Santiago de Chile Chile Oficial de Salud UNICEF Chile, Santiago de Chile.; 2 Facultad de Derecho y Ciencia Política de la Universidad Nacional de Colombia Bogotá Colombia Facultad de Derecho y Ciencia Política de la Universidad Nacional de Colombia, Bogotá, Colombia.; 3 Organismo Andino de Salud – Convenio Hipólito Unanue Lima Perú Organismo Andino de Salud – Convenio Hipólito Unanue, Lima, Perú.; 4 Organismo Andino de Salud – Convenio Hipólito Unanue Lima Perú Organismo Andino de Salud – Convenio Hipólito Unanue, Lima, Perú.; 5 Hospital Luis Calvo Mackenna Santiago de Chile Chile Hospital Luis Calvo Mackenna, Santiago de Chile; 6 Universidad de los Andes Bogotá Colombia Universidad de los Andes, Bogotá, Colombia.; 7 Asesora Despachó del Ministro de Salud y Protección Social Bogotá Colombia Asesora Despachó del Ministro de Salud y Protección Social, Bogotá, Colombia.

**Keywords:** COVID-19, defensa del niño, política pública, cuidado del niño, salud del niño, Chile, Colombia, Perú, COVID-19, child advocacy health policy, public policy, child care, child health, Chile, Colombia, Perú, COVID-19, defesa da criança e do adolescente, política pública, cuidado da criança, saúde da criança, Chile, Colombia, Perú

## Abstract

**Objetivo.:**

Describir las estrategias que fueron establecidas por Chile, Colombia y Perú durante el primer año de la pandemia por COVID-19 y compararlas desde el enfoque de derechos de la niñez.

**Métodos.:**

Se realizó un estudio cualitativo de análisis comparado de políticas públicas, tomando como eje siete categorías construidas por el Capítulo Latinoamericano de la *International-Society-for-Social-Pediatrics-and-Child-Health* a partir de la Convención de Derechos de la Niñez (CDN)*.* La selección de los documentos de los países se realizó por conveniencia y su análisis en diálogos deliberativos.

**Resultados.:**

Se revisaron 173 documentos de los tres países. Destaca como convergencia la priorización de la prevención de la transmisión comunitaria del virus, por sobre la promoción del ejercicio de derechos de la niñez, la falta de participación de niños, niñas y adolescentes (NNA) en el proceso de elaboración de las políticas públicas, y la falta de avance en el reconocimiento y protección del ejercicio de todos sus derechos. No hubo mayores divergencias más allá de brechas de desigualdad identificadas con base a la realidad de cada país.

**Conclusión.:**

La pandemia ha afectado el funcionamiento de los sistemas económicos, sociales, de salud, educación, medioambiente y gobernanza de estos tres países. Si bien este estudio muestra un avance en la inclusión del enfoque de derechos de NNA en las políticas formuladas, su comprensión como sujetos sociales y políticos titulares de derecho podría permitir la construcción de alternativas colectivas que garanticen la salud y el bienestar para todas las personas en el curso de vida.

## Abstract (en)

**Objective.:**

Describe the strategies established by Chile, Colombia, and Peru during the first year of the COVID-19 pandemic and compare them from a children's rights perspective.

**Methods.:**

A qualitative study with comparative analysis of public policies was conducted around seven cat­egories constructed by the Latin American Chapter of the International Society for Social Pediatrics and Child Health, based on the Convention on the Rights of the Child. Country documents were selected based on convenience sampling and were analyzed in deliberative dialogues.

**Results.:**

173 documents from the three countries were reviewed. There was convergence around: prioritization of prevention of community transmission of the virus over promotion of the exercise of children's rights; lack of participation of children and adolescents in the process of developing public policies; and lack of progress in the recognition and protection of the exercise of children’s rights overall. There were no major divergences beyond identified inequality gaps grounded in the reality of each country.

**Conclusion.:**

The pandemic has affected the functioning of the economic, social, health, education, environmental, and governance systems in these three countries. While this study shows progress in the inclusion of the children's rights approach in formulated policies, the recognition of children and adolescents as holders of social and political rights could allow the construction of collective alternatives that guarantee health and well-being for all people throughout the life course.

## RESUMO

**Objetivo.:**

Descrever as estratégias que foram utilizadas por Chile, Colômbia e Peru durante o primeiro ano da pandemia de COVID-19 e compará-las a partir da perspectiva dos direitos da criança.

**Métodos.:**

Realizou-se um estudo qualitativo de análise comparativa de políticas públicas, tomando como eixo sete categorias construídas pelo Capítulo Latino-americano da *International Society for Social Pediatrics & Child Health* a partir da Convenção sobre os Direitos da Criança (CDC)*.* A seleção de documentos dos países foi feita por conveniência, e sua análise ocorreu mediante diálogos deliberativos.

**Resultados.:**

Foram revisados 173 documentos dos três países. A prioridade da prevenção da transmissão comunitária do vírus destaca-se por sua convergência, em detrimento da promoção do exercício dos direitos da criança, da falta de participação de crianças e adolescentes no processo de formulação de políticas públicas e da falta de progresso no reconhecimento e proteção da efetivação de todos os seus direitos. Não houve grandes divergências além das lacunas de desigualdade identificadas com base na realidade de cada país.

**Conclusões.:**

A pandemia afetou o funcionamento dos sistemas econômico, social, de saúde, educação, meio ambiente e governança desses três países. Embora este estudo mostre avanços na inclusão da perspectiva dos direitos da criança e do adolescente nas políticas formuladas, a compreensão deles como sujeitos sociais e políticos titulares de direitos pode permitir a construção de alternativas coletivas que garantam saúde e bem-estar a todas as pessoas ao longo do curso da vida.

Latinoamérica y el Caribe (LAC) ha sido una de las regiones del mundo más afectadas por el coronavirus (COVID-19), representado en el número de casos como de muertes (1). En este contexto, la región enfrenta el complejo desafío de controlar la pandemia sin dejar de garantizar el ejercicio de derechos de su población. La propagación del COVID-19 y sus consecuencias se ven agravados por problemas estructurales internos de los países de la región, como son los elevados niveles de desigualdad, informalidad laboral, pobreza, vulnerabilidad y asentamientos urbanos marginados, así como sus sistemas de salud, educación y de protección social débiles y fragmentados (2-6).

El escaso reconocimiento de niños, niñas y adolescentes (NNA) como sujetos de derechos en las políticas públicas ha dificultado el ejercicio de los derechos consagrados en la Convención de Derechos de la Niñez (CDN). La pandemia y las medidas tomadas por los gobiernos para su control han amplificado aún más esta brecha en función de proteger la salud de la población. Por esto se hace relevante comprender el impacto de la COVID-19 en NNA en la región, considerando un enfoque de derechos de la niñez, entendido como un marco conceptual normativo y operacional -basado en la CDN-, que brinda orientaciones para la promoción y la protección de los derechos en propio articulado, así como mediante las observaciones generales del comité de derechos del niño de la Organización de Naciones Unidas (7-10).

En este esfuerzo, destacan los procesos investigativos generados por la Sociedad Internacional de Pediatría Social (*International-Society-for-Social Pediatrics-and-Child-Health*, ISSOP), que permitieron el intercambio de experiencias y reflexiones desde el comienzo de la pandemia hasta la fecha. Si bien este espacio identificó que los gobiernos, la academia y diversas organizaciones no gubernamentales (ONGs) estaban desarrollando estrategias y acciones para prevenir y dar respuesta a los impactos de la COVID-19 en NNA, no existía hasta la fecha un análisis para entender su impacto en términos del ejercicio de derechos para este grupo. El objetivo de este artículo es describir y comparar las estrategias establecidas por tres países de la región durante el primer año de la pandemia, desde un enfoque de derechos de la niñez, a fin de identificar lecciones a futuro para los países de la Región de las Américas.

## MATERIALES Y MÉTODOS

Se trata de un estudio cualitativo descriptivo de análisis comparado de políticas públicas, (11-13) que contó con una fase de adaptación de la metodología de diálogos deliberativos y otra de análisis de contenido, orientadas por la pregunta: ¿Cuáles son los elementos centrales de la formulación de políticas públicas, con enfoque de derechos de la niñez y adolescencia, en tiempos de pandemia COVID-19?

### Selección de países, estrategia de búsqueda y selección de documentos

Los espacios de diálogo generados por el capítulo de LAC de la ISSOP permitieron intercambios que promovieron la realización de esta investigación. En estas reuniones virtuales de trabajo se presentaba la situación de cada país participante y el impacto que se estaba evidenciando particularmente sobre la salud de NNA. Inicialmente participaron representantes de ocho países de la región quienes aportaron en el análisis de la situación general. Posteriormente, y de acuerdo al interés y disponibilidad de los participantes, Chile, Colombia y Perú se mantuvieron en las sesiones de deliberación con dos investigadores que contaban con capacidad de articular los puntos de vista y las experiencias de los grupos que representaban.

Con el propósito de comprender el sentido del discurso de las estrategias de mitigación de los efectos de la pandemia sobre NNA, y privilegiando el lenguaje escrito (texto) como instrumento para comprender significantes de los tomadores de decisión, se realizó una búsqueda de documentos prescriptivos**,** en sitios web de ministerios, autoridades sanitarias, así como de sociedades científicas o académicas reconocidas en Chile, Colombia y Perú (14-16).

Se consideraron criterios de inclusión: 1) haber sido publicados entre 2020 y marzo del 2021; 2) que reflejaran recomendaciones de tipo prescriptivo para mitigar los efectos de la pandemia por COVID-19 sobre NNA; y 3) que hayan sido escritos bajo el marco de enfoque de derechos humanos. Como único criterio de exclusión se consideró la imposibilidad de acceder al contenido del documento. Los documentos fueron clasificados con base a su carácter y procedencia en: a) gubernamental: normativo y regulatorio; b) promoción, educación y/o difusión de información; c) recomendaciones de la académica, asociación científica o tercer sector; d) documentos con alcance regional.

Los documentos fueron ingresados a una planilla, recopilando nombre, alcance, resumen, número de páginas, enlace de acceso al texto completo, fecha de publicación e inclusión de alguno de los ejes de comparación prestablecidos.

### Diálogos Deliberativos

La metodología de diálogos deliberativo se definen como: ¨mecanismo interactivo para compartir conocimientos [cuyo] objetivo es respaldar la discusión completa de las consideraciones relevantes sobre un asunto de alta prioridad, con el fin de informar la toma de decisiones en políticas y otro tipo de acción [así como] proveer oportunidades para la discusión del problema identificado, las opciones para abordarlo y temas relacionados con barreras, facilitadores o puntos a tener en cuenta respecto a la implementación de aquellas opciones¨ (13). Los encuentros se llevaron a cabo entre los países vinculados al capítulo LAC-ISSOP, entre marzo de 2020 y mayo de 2021, conducido por una facilitadora capacitada quien garantizó que la discusión se mantuviera centrada en los asuntos primordiales y que se escuchasen todas las voces desde una postura crítica. Los pasos llevados a cabo en los 15 diálogos se presentan en el recuadro 1.

### Análisis de contenido

Partiendo de la hipótesis de trabajo derivada de los primeros encuentros: “las políticas públicas para mitigar los efectos de la COVID en NNA de la región se encuadran en un discurso adulto-céntrico” se buscaron, a través del análisis de contenido de los documentos, las ideas expresadas, siendo las unidades de observación el significado de las frases y párrafos, relacionados con la temática (17).

Se comenzó con la lectura de la CDN, a partir de la cuál, en sesión de diálogos, se construyeron siete categorías sobre derechos de la niñez relacionadas con el contexto de la pandemia, presentadas en el recuadro 2.

Una vez definidas las categorías, se realizó a un ejercicio de codificación de base gramatical, llevada a cabo por los investigadores y dirimido por la facilitadora, a fin de confirmar que las categorías fueran homogéneas, exhaustivas, exclusivas, objetivas y adecuadas o pertinentes. Posteriormente, cada uno de los investigadores realizó la codificación de los textos seleccionados por su país, y en caso de diferencias a la hora de comparar codificaciones realizadas, la facilitadora dirimía las mismas. Finalmente, se organizaron los datos en tablas, a partir de las cuales se realizaron inferencias y comparaciones, durante subsecuentes diálogos deliberativos, con el grupo completo de investigadores.

**RECUADRO 1.** Pasos de los Diálogos Deliberativos para la recolección de documentos y análisis de la respuesta con enfoque de derechos de la niñez frente a la pandemia.

Envío por parte de la facilitadora, previo a la reunión, del resumen de la temática, aspecto metodológico o política a analizar En el caso de temática o aspecto metodológico a tratar: exposición por parte de la facilitadora de argumentos de discusión; en el caso de política a analizar: exposición por parte del país del que derivó el documento de características del problema, afectación a NNA y sus familias, alternativas de abordaje y consideraciones clave de implementación Defensa o argumentación de los aspectos clave a incluir o comparar por parte del expositor Deliberación abierta al resto de investigadores, con espacio para contra argumentar Levantamiento de la información a través de resúmenes con disensos o consensos de las discusiones derivadas de cada documento de política

***Fuente***: elaboración propia

**RECUADRO 2.** Categorías construidas sobre los derechos de la niñez afectados por la pandemia de COVID-19.

Estrategias para disminuir o mitigar el impacto sobre la salud física y mental de NNA causadas por las medidas de control de transmisión del COVID-19. Estrategias para identificar y abordar el riesgo psicosocial. Estrategias para evitar la interrupción de cuidados y procesos terapéuticos. Promoción del acompañamiento de las gestantes y de NNA durante el parto y/ u hospitalización Protocolos diferenciados para NNA en situación de marginalidad Participación de NNA Estrategias que aseguren bienes básicos a familias con NNA

***Fuente***: elaboración propia a partir de las categorías construidas

## RESULTADOS

Los tres países difieren en las curvas de contagios, sin embargo, tienen similitudes en las tasas de incidencia de casos COVID-19 en la población general y en NNA, así como la tasa de fallecidos, información relevante para la interpretación de los resultados (cuadro 1).

Se revisaron 173 documentos, 56 de Chile, 58 de Colombia y 59 de Perú, 121 tenían carácter normativo o regulatorio (69.9%); 38 educativos y de difusión de información (19.0%); 15 derivados de asociaciones científicas (8.6%), y 4 de alcance regional, siendo escritos o editados por agencias de las Naciones Unidas (cuadro 2).

A continuación, se presenta una síntesis de la revisión documental categorizada, junto con un análisis conjunto de las estrategias.

### Estrategias para disminuir o mitigar el impacto sobre la salud física y mental de NNA

Los tres países desarrollaron tempranamente estrategias para reducir el contagio de COVID-19, y en distintos tiempos medidas para mitigar sus impactos sobre la salud de NNA. Colombia, a dos meses de iniciada la pandemia, presentó una estrategia para el cuidado de la salud de NNA, con recomendaciones para brindar acompañamiento socioemocional en casa. Priorizaron a NNA con condiciones de salud mental en desescalonamiento del aislamiento. En Chile, al cuarto mes de iniciada la pandemia se lanza el plan “paso a paso”, con medidas y excepciones para NNA del espectro autista y trastornos conductuales, y se elaboró la Guía Práctica de Bienestar Emocional y la plataforma *SaludableMente* para apoyar su salud mental y el bienestar psicosocial. Perú estableció excepciones al desplazamiento de NNA fuera del domicilio en tiempos de confinamiento al segundo mes de la pandemia. Se elaboró la Guía-técnica-para-cuidado-de-la-salud-mental con capítulos específicos de NNA, y el Plan de Salud Mental en el contexto COVID-19.

Los tres países establecieron medidas de confinamiento y restricción de desplazamiento como cierre de escuelas y espacios públicos. Sin embargo, dispusieron franjas horarias en las cuales se podía salir a espacios abiertos, durante la cuarentena. Los tres países desarrollaron estrategias de apoyo socioemocional de NNA y sus familias y dispusieron, en distintos tiempos, canales informativos para la prevención de la enfermedad.

### Estrategias para identificar y abordar riesgo psicosocial

Los tres países establecieron acciones para prevenir y denunciar el maltrato infantil. En Chile, se crearon canales de comunicación específicos (FonoNiños-147 de Carabineros de Chile para NNA víctimas de vulneraciones de derechos; FonoFamilia-149 para orientación y auxilio a familias; FonoInfancia para apoyo psicológico de la crianza; y LíneaLibre-1515 para brindar orientación psicológica a NNA desde ONGs). Se implementó la campaña ciudadana “Mascarilla-19” para atender a mujeres víctimas de violencia intrafamiliar. En Colombia, se publicaron orientaciones para prevenir las violencias contra NNA ofreciendo redes de apoyo (REDPAPAZ) y acceso a información institucional virtual. Se publicaron lineamientos para la atención de salud de NNA con COVID-19, con apartados para la prevención y detección de riesgo psicosocial estableciendo signos de alarma para su atención inmediata e integral. En Perú, el Ministerio de la Mujer y Poblaciones Vulnerables, fortalecieron canales de denuncia frente a situaciones de riesgo o desprotección familiar de NNA y establecieron mecanismos para garantizar el ejercicio de sus derechos. Se aprobó el Reglamento de Mecanismos de Prevención de Violencia en la Familia, y se publicaron los Protocolos para la Gestión de la Convivencia Escolar la Prevención y Atención de la Violencia contra NNA. Además, la Defensoría del Pueblo elaboró informes especiales sobre la materia.

**CUADRO 1.** Indicadores demográficos y epidemiológicos relacionados con la COVID-19 en Chile, Colombia y Perú

País	Chile	Colombia	Perú
Población	18,7 millones de habitantes	51.2 millones de habitantes	34,1 millones de habitantes
Fecha del Primer caso COVID-19	3 de marzo, 2020	6 de marzo, 2020	6 de marzo, 2020
Número de contagios*	864 000	2 273 245	1 371 176
Número de contagios en menores de 19 años*	69 500	170 201	110 197
Casos confirmados por 100 000 habitantes*	4 500	4 440	4 019
Casos confirmados por 100 000 habitantes menores de 19 años*	1 800	1 072	978
Número de fallecidos*	28 686**	60 412	47 854
Número de fallecidos en menores de 19 años*	265**	306	287
Fallecidos por 100.000 habitantes*	110	118	140

* a 1 año del primer caso;

** Confirmados + sospechosos;

***Fuente***: elaboración propia a partir de las fuentes oficiales de cada país reportadas por los Ministerios de Salud.

**CUADRO 2.** Características de los documentos revisados por país.

	Chile	Colombia	Perú	Total
**Totalidad de documentos aportados Número (%)**	**56** **(32,3%)**	**58** **(33,5%)**	**59** **(34,1%)**	**173**
Documento nacional con carácter normativo y regulatorio.	40	41	40	121
Documentos de promoción, educación y difusión de información.	11	8	40	33
Recomendaciones de la academia, asociación científica o tercer sector.	5	6	4	15
Documentos con alcance regional: UNICEF, OIM, OPS/OMS.	0	3	1	4

***Fuente***: elaboración propia a partir de los resultados obtenidos

En los tres países se crearon canales de comunicación y respuesta para la denuncia de problemas de riesgo psicosocial, así como redes de apoyo. Sobre este punto en los diálogos deliberativos hubo convergencia, en que existe un silencio respecto a sistemas de monitoreo activo a las violencias (que se han visto incrementadas durante la pandemia) que demuestren la protección efectiva de los casos identificados.

### Estrategias para evitar la interrupción de cuidados y procesos terapéuticos

En los tres países se establecen estrategias para evitar la interrupción de cuidados y procesos terapéuticos, priorizando los más críticos. Hay convergencia en la priorización del control presencial de gestantes, recién nacidos y lactantes, programas de inmunizaciones, y continuidad a la atención en planificación familiar. En Chile se publicó el protocolo para prevención de transmisión de COVID-19 en unidades de pediatría y de cuidados intensivos, promoviendo la hospitalización acompañada. Perú publicó un decreto supremo para garantizar las prestaciones de prevención y control de la anemia; normas referentes al Plan de Recuperación de Brechas en Inmunizaciones y Anemia y medidas para incrementar su capacidad de respuesta de autoridades locales de salud. En relación al manejo del COVID-19 y sus presentaciones graves, los tres países desarrollaron protocolos de atención, así como protocolos para el manejo del síndrome inflamatorio multisistémico pediátrico.

En los diálogos deliberativos hay concordancia sobre la necesidad de evaluar el funcionamiento de dichas estrategias y su efectividad en la mitigación efectiva del impacto de la COVID-19 y la pandemia sobre la salud de NNA.

### Promoción del acompañamiento de las gestantes durante el parto y de la hospitalización de NNA

Todos los países establecieron protocolos de acompañamiento en el parto, pero en distintos tiempos. En Chile, se establecieron protocolos de acompañamiento al quinto mes del primer caso. Al sexto mes se publicó la orientación técnica que recomienda el acompañamiento de NNA en casos específicos como lactantes y NNA con trastornos de la conducta o del espectro autista. En Colombia, se elaboró el protocolo de acompañamiento en el parto, al tercer mes del primer caso de COVID-19 y en el Perú, se promulgó el protocolo de acompañamiento en el parto, al cumplir un mes del primer caso. Sobre el acompañamiento de NNA hospitalizados, hay menos información. Todos los protocolos establecen normativas para garantizar un acompañamiento seguro para todos los involucrados, y para gestionar adaptaciones al espacio físico según la realidad local.

### Protocolos diferenciados para NNA en situación de marginalidad

En Chile, el Subsistema de Protección Integral de la Infancia Chile Crece Contigo, estableció estrategias para acercar los servicios sociales a la comunidad. Los Ministerios de Salud, Educación y Desarrollo Social y Familia (MDSF) elaboraron material en los idiomas de pueblos originarios e inmigrantes. El Servicio Nacional de Discapacidad, con agrupaciones de la sociedad civil, pacientes y academia, elaboraron materiales que brindan información a personas con discapacidad sobre sus derechos, y recomendaciones a los equipos de salud para brindar atención segura a estas poblaciones. El MDSF también estableció estrategias para disminuir el riesgo de contagio en población migrantes y familias en situación de pobreza. En Colombia, se elaboraron protocolos para facilitar la identificación de NNA en el Sistema de Responsabilidad Penal Adolescente (SRPA) y a aquellos desvinculados del conflicto armado, y su afiliación al sistema de seguridad social. Se dispusieron lineamientos para la prevención, contención y mitigación de COVID-19 y atención de salud en adolescentes del SRPA y migrantes. En el Perú, se aprobó el decreto que establece acciones para la protección de los pueblos originarios, con atención prioritaria a NNA; también se establecieron lineamientos para identificar casos sospechosos de COVID-19 en pueblos indígenas y afroperuanos para realizar seguimiento y monitoreo durante el tratamiento. Fue elaborado material educativo en lenguas originarias y se crearon normas para enmendar el hacinamiento en establecimientos penitenciarios con medidas socioeducativas para adolescentes privados de libertad. Se entregaron tabletas con internet a instituciones educativas públicas con prioridad en alumnos en situación de pobreza. Se brindó atención alimentaria complementaria y se aprobaron los modelos de “Atención Oportuna de NNA en presunto estado de abandono”.

Los tres países presentaron estrategias focalizadas para mejorar el acceso a servicios sociales y de salud de la población y de NNA en situación de marginalidad (discapacidad, migración, grupos étnicos, entre otros).

### Participación de NNA

En Chile, el Ministerio de Salud abrió espacios de diálogo con NNA para reflexionar sobre el cierre de las escuelas y el impacto de la restricción de desplazamiento. Si bien se recogió sistemáticamente su opinión, no queda claro si fueron considerados en la toma de decisiones por parte de la autoridad. La Sociedad Chilena de Pediatría y la Defensoría de la Niñez generaron espacios de participación y publicaron información levantada para movilizar a los tomadores de decisión a favor de la niñez. En el Perú, se han mantenido activos espacios de participación de NNA como el Colectivo Interinstitucional por los Derechos de la Niñez y Adolescencia, el Consejo Consultivo de NNA, y la Mesa de Concertación para la Lucha Contra la Pobreza. Se han realizado foros y conversatorios intergeneracionales sobre protección de derechos en el contexto de COVID-19 por diversas organizaciones. Se destaca también el Organismo Andino de Salud - Convenio Hipólito Unanue con actividades con NNA para promover el autocuidado y elaborar la sistematización que llevó por título: *“¿Qué dicen los NNA sobre la vacunación contra COVID19 en Perú?”* Aunque la participación es un asunto prioritario en el marco normativo general de Colombia, en la revisión documental no se reportaron estrategias de políticas actualizadas de participación infantil en el marco de la COVID-19.

### Estrategias que aseguren bienes básicos

Esta es la categoría con más divergencias, principalmente por las problemáticas particulares de cada país. El elemento transversal fueron los apoyos vía transferencias directas o subsidios de distintos montos y a distintos tiempos. En cuanto a seguridad alimentaria de NNA, en Colombia, se dictaron medidas que brindan herramientas a las entidades territoriales para garantizar la ejecución del Programa de Alimentación Escolar (PAE) a todas las instituciones educativas públicas del país. En Perú se entregaron canastas de alimentos para más de 2,5 millones de hogares, al igual que en Chile en que el alimento que se daba en las escuelas se entregó como canastas familiares.

En los tres países se establecieron disposiciones para fraccionamiento de pago de servicios básicos, se adelantaron las transferencias de programas sociales y hubo provisión excepcional de alimentos para población en situación de pobreza. En Perú se brindó asistencia económica para NNA cuyos cuidadores hayan fallecido por COVID-19. En Chile, tras importantes demandas ciudadanas, el poder judicial sentenció al Estado para que asegurara 100 litros de agua potable por día por persona y el apoyo económico ha sido por medio de tres mecanismos: retiros de los propios ahorros de los fondos de pensiones; la entrega de un ingreso familiar de emergencia al 60% de las familias con mayor necesidad económica del país; y un préstamo estatal solidario a los individuos que lo solicitaron.

En los tres países se evidenciaron brechas de acceso a servicios básicos, saneamiento, emergencia de trabajo informal, trabajo infantil entre otros problemas sociales, a los que se han dado respuestas coyunturales, principalmente por políticas focalizadas.

## DISCUSIÓN

La investigación identifica y contrasta elementos presentes o ausentes en la formulación de las políticas públicas establecidas para enfrentar la pandemia, desde una perspectiva de derechos de la niñez. Corresponde señalar como limitación la selección de países participantes por conveniencia, lo que no permite tener una visión amplia y acabada de lo que ocurre en la región. Respecto a la estrategia de búsqueda, si bien no se basó en una revisión sistemática, ésta cumplió con chequeo de pares y verificación externa. A su vez es necesario destacar que, dado el curso incierto de la pandemia, toda conclusión es preliminar y parcial. Teniendo en cuenta que este estudio analiza las bases discursivas de las políticas públicas formuladas, se consideraría adecuado un nuevo análisis comparativo, en caso de que se observe un giro en base epistemológica de los enfoques de las políticas públicas.

La pandemia por COVID-19, así como la respuesta establecida por los gobiernos han interferido ampliamente con las actividades humanas generando amplios efectos en los sistemas de políticos, económicos y sociales, en todo el mundo (18). Los resultados presentados en este estudio, al igual que publicaciones de otras regiones del mundo sugieren que el reconocimiento y protección de los derechos de la CDN se han visto importantemente restringidos (19). Algunos derechos intentaron ser restituidos por vías alternativas con resultados parciales como el aumento de las brechas de trayectorias educativas por nivel socioeconómico (20), género (21) y la conectividad (22), como consecuencia de la impartición de educación virtual en diversos países de la región (23). Si bien esta dimensión no fue profundizada en este estudio, apareció transversalmente en todas las categorías.

El aumento de las brechas sociales y la exacerbación de situaciones de vulnerabilidad se tradujeron en la elevación de contagios y letalidad en poblaciones específicas. El acompañamiento a dichas familias, la continuidad de los servicios y el fortalecimiento de la protección social fueron claves para el desarrollo de la resiliencia comunitaria (24). Considerando la inequidad de la región, se recomienda que los gobiernos implementen medidas efectivas para disminuir el contagio y tratar la enfermedad, sin descuidar el proceso salud-enfermedad, impulsando modelos de cuidado y atención a las familias de base comunitaria, así como su participación en la toma de decisiones (25).

Respecto al derecho a ser oídos, Perú y Chile sobresalieron, ya que, a través de consejos consultivos de NNA, han contribuido al avance hacia formas de participación auténtica, superando los tres primeros peldaños -manipulación, decoración y participación simbólica- de la escala de Hart (26). Aunque Colombia no reportó estrategias actualizadas en respuesta a la COVID-19, éste destaca por sus políticas de participación social en salud (27).

En relación al análisis discursivo, la principal convergencia muestra que, a pesar del temprano consenso científico respecto al rol menor de los NNA en la propagación del virus, las estrategias se focalizaron en restricciones de desplazamiento y prolongados cierres de espacios educativos y recreativos fundamentales para el desarrollo de NNA, con sus deletéreas consecuencias (28, 29). En diálogo con estudios que afirman que las políticas establecidas y sus modos hegemónicos de formulación no son circunstanciales, sino estructurales (30), nuestros resultados sugieren que las políticas públicas analizadas están enmarcadas en enfoques convencionales, los cuales buscan resolver problemas desde comprensiones adultocéntricas, lineales, técnico científicas y con escasa participación de los sujetos a quienes van dirigidas, reproduciendo así las inequidades del modelo civilizatorio epocal (31). Al respecto, el análisis muestra la necesidad de que los países consideren a los NNA como sujetos sociales y políticos, e innoven en maneras alternativas para su participación en los asuntos que les competen (25, 32). Los enfoques interpretativo-críticos de análisis de políticas públicas, la medicina social y la salud colectiva (33), con teorías latinoamericanas específicas como la epidemiología crítica y la determinación social en salud podrían ser referentes para avanzar en nuevas formas de elaborar políticas públicas en salud (30,32-35).

En conclusión, el análisis confirma la necesidad de que los países consideren a los NNA como sujetos de derecho en el ciclo de las políticas públicas. Con la pandemia por COVID-19, ha quedado demostrado que tanto las características de los países como los enfoques imperantes de análisis de políticas públicas en salud requieren poner especial atención a la participación deliberativa de los NNA. Los resultados también pueden ser útiles para otros países de la región o del mundo, para el enfrentamiento de otras situaciones en donde los derechos entren en conflicto. El ejemplo del enfrentamiento de la pandemia por VIH/Sida y su tránsito desde un abordaje biomédico asistencial a uno biopsicosocial con enfoque de derechos, podría servir como referente en la coyuntura sanitaria y social actual, particularmente desde una mirada de los derechos de la niñez. Abordar los efectos sociales a largo plazo de la pandemia, incluido el deterioro de la salud mental, las inequidades y la pobreza, serán temas centrales para la resiliencia social en la fase de recuperación.

## Declaración.

Las opiniones expresadas en este manuscrito son responsabilidad del autor y no reflejan necesariamente los criterios ni la política de la *RPSP/PAJPH* y/o de la OPS.
